# Characterization of Platinum-Based Thin Films Deposited by Thermionic Vacuum Arc (TVA) Method

**DOI:** 10.3390/ma13071796

**Published:** 2020-04-10

**Authors:** Sebastian Cozma, Rodica Vlǎdoiu, Aurelia Mandes, Virginia Dinca, Gabriel Prodan, Vilma Buršíková

**Affiliations:** 1 Department of Physics, Faculty of Applied Sciences and Engineering, Ovidius University of Constanta, Bvd. Mamaia, No 124, 900527 Constanta, Romania; sebastian.cozma@protonmail.com (S.C.); rodicavladoiu@gmail.com (R.V.); amandes@univ-ovidius.ro (A.M.); gprodan@univ-ovidius.ro (G.P.); 2 Institute of Physical Electronics, Faculty of Science, Masaryk University, Kotlarska 2, 611 37 Brno, Czech Republic; vilmab@physics.muni.cz

**Keywords:** Pt, PtTi thin films, TVA, morphological properties

## Abstract

The current work aimed to characterize the morphology, chemical, and mechanical properties of Pt and PtTi thin films deposited via thermionic vacuum arc (TVA) method on glass and silicon substrates. The deposited thin films were characterized by means of a scanning electron microscope technique (SEM). The quantitative elemental microanalysis was done using energy-dispersive X-ray spectroscopy (EDS). The tribological properties were studied by a ball-on-disc tribometer, and the mechanical properties were measured using nanoindentation tests. The roughness, as well as the micro and nanoscale features, were characterized using atomic force microscopy (AFM) and transmission electron microscopy (TEM). The wettability of the deposited Pt and PtTi thin films was investigated by the surface free energy evaluation (SFE) method. The purpose of our study was to prove the potential applications of Pt-based thin films in fields, such as nanoelectronics, fuel cells, medicine, and materials science.

## 1. Introduction

Platinum (Pt) is considered as a promising material for industrial applications due to its unique properties, such as corrosion resistance, high electronic conductivity, thermal stability, excellent chemical stability, high catalytic activity, and good solubility for hydrogen and oxygen. The noble platinum thin films have found numerous applications in the microelectromechanical systems (MEMS), jewellery industry, watch industry, and automotive industry. However, the main applications of Pt thin films are found in the automotive industry, where it is used in catalytic converters for vehicle emissions control devices. Pt coatings are also extensively used for electrodes, high-end spark plugs coatings, oxygen sensors, and turbine engines [1,2,3,4,5,6,7].

One of the main disadvantages of platinum films is the poor adhesion of the layers on silicon dioxide and silicon nitride because Pt easily reacts with silicon and forms a platinum silicide. The total interfacial and surface energy of Pt thin film can be minimized by reducing the ceramic-metal contact area by creating islands of Pt material. However, this leads to a loss of electrical conductivity and a considerable decrease in functionality, reliability, and sensitivity of MEMS [8,9,10]. Further, determination and control of residual stress in Pt thin films is important for industrial applications. In spite of their importance, residual stresses are difficult to foresee, and there are only a few studies [11,12]. Due to the increased markets for these areas, the demand for this metal and its price have greatly increased, and, because of very limited sources, this metal is rated as critical.

More efficient use of platinum thin films means a reduction of the quantity of the material in an application, but still, ensuring the same function of the application. For this reason, the substitution of this metal in its applications would be useful, but because of its famous properties, this is very difficult [13]. One of the solutions is to develop new materials with different content of platinum to improve the properties of thin films [14].

Hence, adhesion can be enhanced by intermediate titanium (Ti) protective coating between the Pt film and the substrate or including the Ti into the Pt matrix. This is supported by the fact that these two metals exhibit quite similar melting points: T_Ti_ = 1941 K and T_Pt_ = 2041.4 K. At the same time, the state of surfaces at operating conditions of these components becomes more important due to the development and miniaturization of device components.

In the scientific literature, the synthesis of platinum thin films by magnetron sputtering, electron beam evaporation, wet impregnation, thermal evaporation pulsed laser deposition, and atomic layer deposition (ALD) is already studied. Most of the prepared films are polycrystalline with different defects, while the physical and electrochemical characterization of deposited films remains limited [15,16,17]. 

In this paper, the thermionic vacuum arc method was used to obtain a combinatory metal (Ti) inclusion in the Pt matrix that promotes the benefits of Pt and reduces their secondary reactions for industrial applications. The original thermionic vacuum arc (TVA) method [18], as a deposition process for platinum-based nanocomposite thin films, might become one of the most suitable technologies to create materials with tailored grain size and composition, with applications in nanoelectronics and medicine.

## 2. Experimental Setup 

### 2.1. Deposition Method 

The TVA deposition method consists of evaporating the solid material placed in a crucible (the anode) by electron bombardment. The electrons are ejected from a tungsten filament by heating it via external circuitry. By using a Wehnelt cylinder and having a high voltage drop on the anode, the emitted electron beam can be accelerated and focused on the target material.

The electrodes are placed in an ultra-high vacuum (UHV) chamber in order to maximize the electron mean free path and, therefore, the electron energy. The external cathode heating circuitry can easily generate an intense electron flux, which increases the vapor pressure in the space between the electrodes, obtaining a discharge plasma by ionizing the particles of the evaporated material. Thus, the generated plasma expands in the vacuum chamber, touching the substrate [19,20,21,22,23]. This generated plasma minimizes the contamination of the deposited thin film, increases its structural quality due to the presence of plasma ions that can be accelerated to the adequately polarized substrates and, due to using electrons instead of ions to vaporize the material [24,25,26].

The evaporation systems between the electrodes behave like point sources, placed in certain positions in the vacuum chamber. The absence of a filling gas in the chamber contributes to a minimum of inclusions in the obtained film, and the low surface of the generated plasma allows the use of multiple anodes [27].

### 2.2. Experimental Parameters

The experimental device consists of the deposition chamber and an active water cooling system. The detailed figure of the used configuration is described elsewhere [28]. The heat is transferred to the coolant in the metallic alcoves placed tangent to the deposition chamber.

Pt and PtTi thin films were deposited on glass and silicon substrates by the TVA method at a starting pressure of *p = 3.5 × 10^−4^ Pa* in the vacuum chamber, having a filament current intensity of *I_f_ = 54 A*. In both cases, the plasma discharge started at a cathode voltage drop of *V_Pt_ = 3.5 kV* and a cathode current intensity of *I_Pt_ = 199 mA*. After deposition, the pressure stabilized at a value of *p_Pt_ = 2.66 × 10^−2^ Pa*. These experimental parameters for Pt deposition were different from those presented in [14], in terms of procedure and final thickness of the Pt on the thin film, according to the application for fuel cells. 

The total deposition time was *t_d_ = 480 s*, and the distance between the substrate and the plasma discharge was *d = 8 × 10^−3^ m*. The resulting film thickness as measured by a Cressington thickness monitor device, attached to the deposition chamber, was *t = 10^−7^ m*. 

### 2.3. Surface Structural Analysis and Elemental Analysis

To characterize the surface of the deposited films, we used a scanning electron microscope (SEM) of the type EVO 50 XVP Carl Zeiss NTS fabricated in Jena, Germany. The elemental analysis was done by using energy-dispersive x-ray spectroscopy (EDS) attachment from Bruker (Billerica, MA, USA). For SEM investigation, a special glue fixed the samples on the round stage of the microscope.

### 2.4. Mechanical Properties Characterization

The Hysitron TI950 (Bruker, Hysitron, MN, USA) dual-head nanoindenter equipped with a sharp Berkovich diamond tip (with a tip diameter around 50 nm) was used to measure the hardness of Pt and PtTi films. The tip was calibrated immediately before the hardness measurement using a certified fused silica substrate, and quasistatic partial-unload (QSpul) mode with 20 unloading segments (9 measurements) was used in the range from 0.5 to 10 mN to measure the depth dependence of the hardness and the elastic modulus of the films deposited on glass substrates.

The effective elastic modulus *E*_eff_ was calculated from the measured reduced elastic modulus *E_r_* according to the following formula:

(1) $$\frac{1}{E_{r}}=\frac{1-v^{2}}{E}+\frac{1-v_{i}^{2}}{E_{i}}=\frac{1}{E_{eff}}+\frac{1-v_{i}^{2}}{E_{i}},$$

Here *E_i_* and ν_i_ are Young's modulus and Poisson ratio of the diamond indenter; *E* and *ν* are the same characteristics of the studied sample, respectively [29]. 

The roughness and the micro and nanoscale features were determined via atomic force microscopy (AFM), using a device of the Ntegra Prima (NT-MDT, Moscow, Russia) type for the 3D characterization. The dynamic semi-contact (“tapping” or “intermittent”) mode [30] was used with a scanning rate of 0.5 Hz and a resolution of 512 × 512. The studied samples were glued to metal targets and fixed using the magnetic stage of the microscope. The advantage of this mode was that in addition to topography maps, it also enabled to map the phase and the magnitude of the tip oscillation during the surface scanning. The phase lag between the external excitation of the oscillating tip and its response to the tip-surface interactions was related to the local energy dissipation on the surface. This local energy dissipation could be related to local material property changes of the mapped surface. For example, it gave the opportunity to clearly visualize the grains and boundaries of the sample. The magnitude map (feedback error map) was achieved using the mapping of the cantilever oscillation amplitude, which is usually achieved faster than the preset value of the cantilever oscillation amplitude reached by the feedback system. This map contains some additional information about the topography, and it can be utilized for achieving a more precise illustration of the surface relief.

To characterize the tribological properties of the thin films’ surfaces, we determined the friction coefficient using a ball-on-disk tribometer made by CSM Switzerland. 

### 2.5. Surface Free Energy (SFE) Evaluation

The surface free energy determines the wettability of a certain surface. An experimental method of evaluating this property is by determining the contact angle, θ (the angle between the gas–solid interface and the solid–liquid interface). 

In order to evaluate the wettability of the deposited thin films, the SEE System (surface energy evaluation system) device was used, with its corresponding SEE Software 6.0. The used control liquids were water and ethylene glycol; the whole evaluation was done under standard temperature and pressure (STP) conditions.

### 2.6. Surface Morphology and Structure Investigation

The investigation of the film microstructure was acquired using Phillips CM 120 ST (Phillips, Amsterdam, Netherlands) transmission electron microscope (TEM) provided with HR-TEM (high resolution-transmission electroscope microscopy) and selected area electron diffraction (SAED) facilities. The morphology of the Pt/Si and PtTi/Si samples was studied by estimating Feret’s diameter of the projected area of grain in TEM images. 

## 3. Results and Discussion

### 3.1. SEM/EDS Analysis 

**Figure 1:**
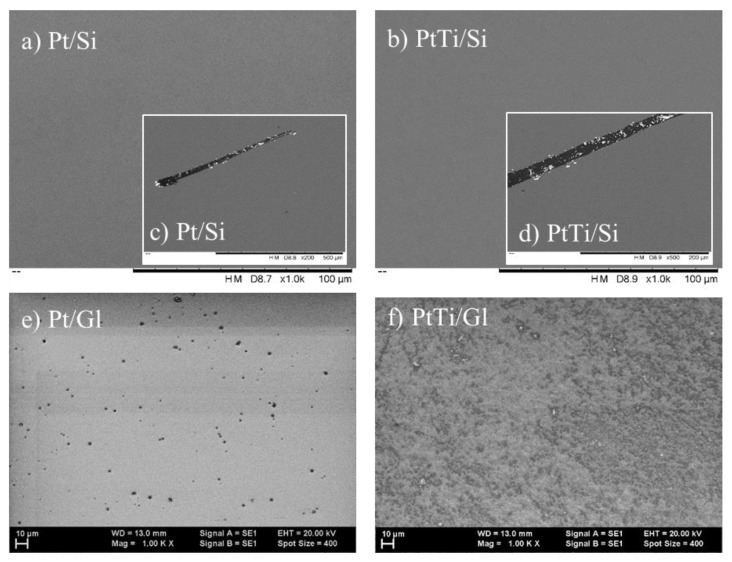
SEM images of: (**a**) Pt/Si and (**b**) PtTi/Si thin films deposited on Si substrate at an acceleration voltage of 15 kV and magnifications of: 1000×, and SEM images after the scratch for the determination of adhesion test: (**c**) Pt/Si and (**d**) PtTi/Si film. Images (**e**) and (**f**) present Pt/Gl and PtTi/Gl thin films deposited on a glass substrate.

The SEM images of the Pt and PtTi thin film deposited on Si substrate showed great layer uniformity, a considerable purity without noteworthy inclusions or surface defects (Figure 1). 

In the case of Pt and PtTi thin film deposited on a glass substrate, the SEM images showed also great uniformity but also some inclusions with diameters up to 2 µm, superficial holes (diameters up to 3 µm), and particles trapped between the substrate and the deposited material with diameters ranging from 300 nm to 1 µm. The SEM images of the PtTi thin film deposited on silicon surface showed uniformity, with the exception of a single 200 µm-thick trace from adhesion analysis. The EDS analysis for Pt thin film (Table 1) showed a Pt to total weight ratio of 55.14 wt.%, and the normalized atomic percentage was 18.02 at.%.

**Figure 2:**
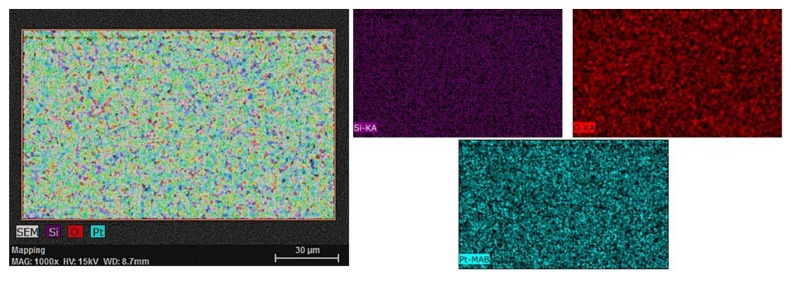
Graphical elemental mapping for Pt thin film deposited on Si substrate at 1000× magnification and 15 kV acceleration voltage.

From the elemental mapping (Figure 2), we could identify the main impurities as being oxygen as traces in the deposition chamber.

**Figure 3:**
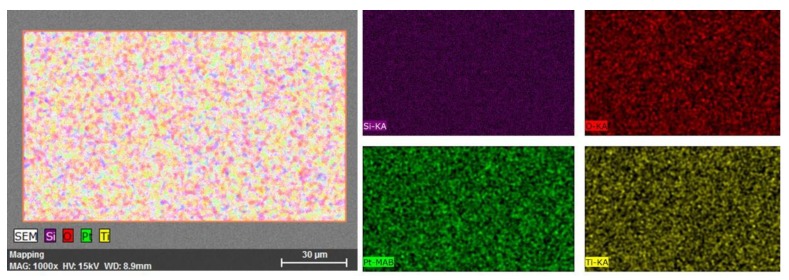
Graphical elemental mapping for PtTi thin film deposited on a glass substrate at 1000× magnification and 15 kV acceleration voltage.

Ti could be seen in the graphical elemental mapping (Figure 3), realized at 1000× magnification.

The Pt total weight ratio was 57.01%, and the Ti was 9.57%. The normalized atomic percentage for Pt was 34.33% and 7.06% for Ti (Table 2). 

In both samples, the presence of oxygen could be attributed to the traces that remained in the vacuum chamber.

### 3.2. Friction Coefficient

The friction coefficient of Pt and PtTi thin films deposited on the glass substrate was determined using a ball-on-disk tribometer. The steel ball was pressed against the surfaces of the thin films with forces of 0.5 N, 1 N, and 3 N. Each measurement was made for a traveled distance of 20 m, at room temperature, and relative humidity of 40%. The average friction coefficient, as well as the distance traveled by the ball before reaching the substrate, are presented in Table 3.

**Figure 4:**
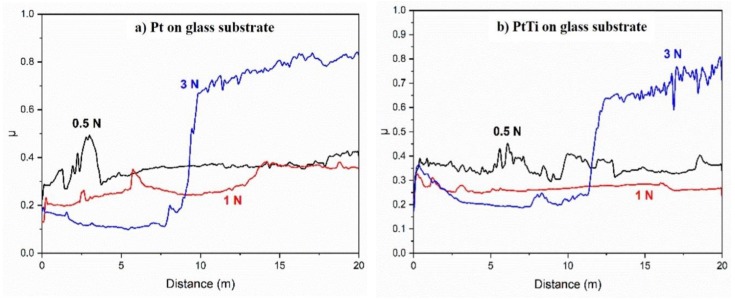
Overlapped graphs of friction coefficient as a function of the distance at an applied force of 0.5 N, 1 N, and 3 N, for (**a**) Pt on a glass substrate and (**b**) PtTi on a glass substrate.

The graphs in Figure 4 present the friction coefficients as functions of the traveled distance for both materials and all three applied forces. 

By adding even a relatively small amount of Ti (20%) in the Pt thin film, the coefficient of friction was decreased. This could be a promising combination for applications where the enhanced tribological properties are required.

### 3.3. Mechanical Properties

**Figure 5:**
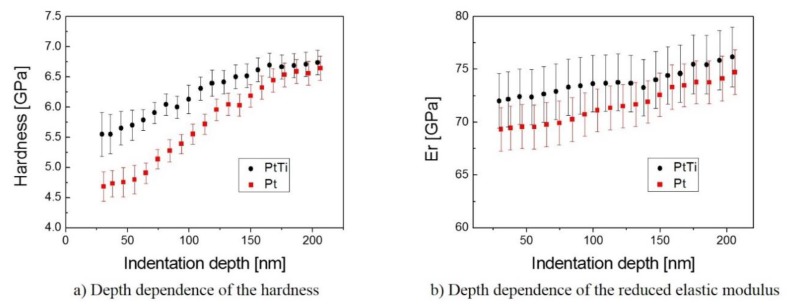
Results of the nanoindentation tests in the range of indentation loads from 0.5 to 10 mN. (**a**) Depth dependence of the hardness and (**b**) Depth dependence of the reduced elastic modulus.

The mechanical properties of the samples were studied by nanoindentation using the QSpul method in order to study the response of the film-substrate system from the near-surface through the film-substrate interface up to the glass substrate. Each unloading segment of the QSpul force-displacement curve was evaluated using the method of Oliver and Pharr [29]. The QSpul method made it possible to determine the interval of indentation depths, where the measured data represent the film properties, and the interval of indentation depths, where the measured data are already affected by the substrate. According to FEM results in [31], for soft films on harder substrates, the measured film hardness value is not affected by the substrate hardness, substantially up to an indentation depth equal to half the film thickness. Contrary to the hardness, the elastic modulus exhibits a more sensitive response to the substrate elastic properties with the increasing indentation depth. In the case of the film with a lower elastic modulus compared to the substrates, the indentation depth should be around one-tenth of the film thickness in order to get the correct value of the elastic modulus. According to work described in [29], the hardness results obtained around indentation depths of 50 nm represent the film hardness, and, for the results obtained from 150 nm of indentation depth, the influence of the substrate material (glass) is dominant [32]. The film elastic properties might be estimated on the bases of the reduced elastic modulus values obtained at indentation depths lower than 50 nm. The results from indentation tests were in good accordance with the results of the film wear resistance (see the distance traveled before reaching the substrate in Table 3) obtained using the tribological measurements. The PtTi film showed significantly higher hardness (5.5 ± 0.1 GPa) than the Pt film without Ti content (4.7 ± 0.1 GPa). However, their elastic modulus differed only slightly (see Figure 5).

### 3.4. AFM Analysis

The surface roughness of the thin films deposited on a glass substrate, as well as the micro and nanoscale features, were determined via atomic force microscopy (AFM) (for 3D characterization). The AFM operated solely in tapping mode, enabling not only topography mapping but also mapping the phase shift between the external excitation of the oscillating tip and its response to the tip-surface interactions and the difference between the preset and instantaneous amplitude (feedback error) of the tip oscillation (see Section 2.4). The last two methods gave the opportunity to visualize grains, boundaries, and the relief of the sample surface more precisely than in the case of the simple topography map.

**Figure 6:**
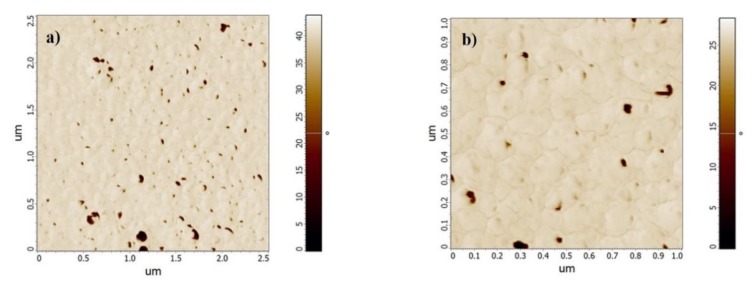
Phase maps for Pt deposited on a glass substrate for areas of (**a**) 2.5 × 2.5 µm and (**b**) 1 × 1 µm.

For the Pt thin film on a glass substrate, the average roughness was R_a_ = 3.8 nm. According to the phase maps, the film consisted of nanograins with a diameter of approx. 100 nm, indicated by the phase contrast maps in Figure 6. 

**Figure 7:**
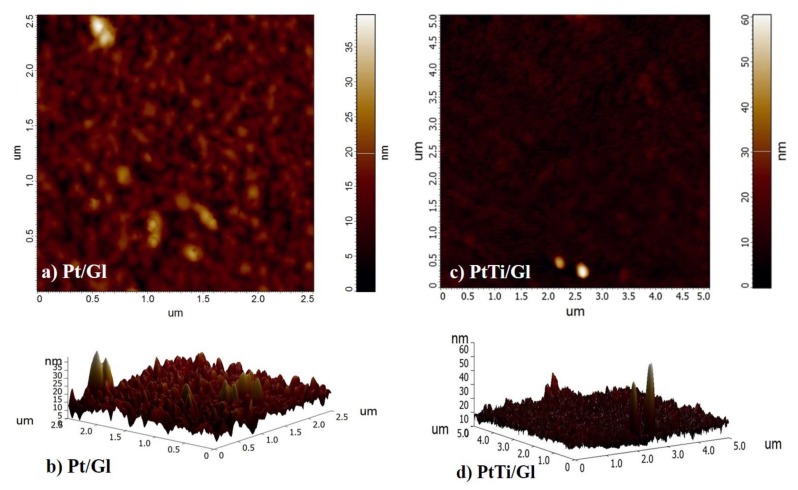
2D (**a**) and 3D topography map (**b**) for Pt; 2D (**c**) and 3D topography image (**d**) for PtTi thin film. The studied films were deposited on a glass substrate.

In the case of PtTi, the average roughness was determined as being R_a_ = 8.8 nm (Figure 7).

Having said that the deposited layer thickness was about 100 nm, the roughness values, presented in the Table 4, were negligible for non-critical applications (e.g., semiconductors, reference parts, etc.). The peaks shown in the AFM 3D profile for the PtTi film were due to surface impurities and could be mitigated using surface treatment processes.

### 3.5. Wettability

**Figure 8:**
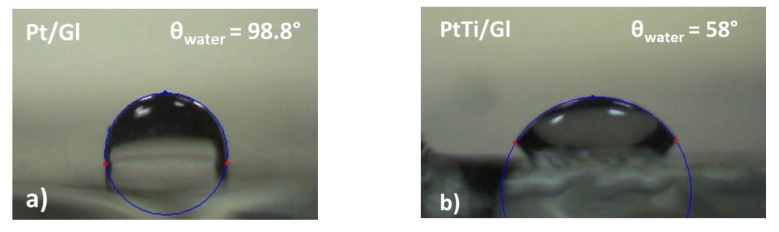
Droplet images and the calculated contact angles by SEE system software using water on a glass substrate: (**a**) of Pt and (**b**) of PtTi thin films.

By using SEE System, we managed to determine the hydrophilicity or hydrophobicity of the surfaces of the deposited films. The measurements were carried out in air at room temperature for Pt and PtTi on glass substrates, using a sessile drop method and 2 µL deionized water droplets. For each sample, the contact angles were measured five times, and the average values were adopted (Figure 8).

Therefore, as indicated by the contact angles, the thin films deposited on glass substrate had different behaviors: hydrophobic, with an average contact angle for the water of *θ_H2O(Gl)_ = 98.8°* θH2O(Gl)=98,8° for Pt and hydrophilic with *θ_H2O(Gl)_ = 58°* for PtTi on a glass substrate.

The oxygen concentration in the substrate seemed to decrease the contact angle, thus increasing the hydrophilicity of the film. The same applied while using ethylene glycol as a control liquid. The values of the free surface energy (SFE) evaluated by the Wu-Equation of State model were the following: for Pt: *γ _Pt/Gl_ =* *13.20 mJ/m^2^* and for PtTi/Gl: *γ _PtTi/Gl_ =* *42.60 mJ/m^2^*. The calculation procedure could be found in Ref. [33].

It is also worth noting that surface oxidation would inevitably be produced on the Ti surfaces, particularly on the PtTi surfaces with higher surface areas. From the EDS analysis, it was clearly revealed that both films were composed of oxygen, indicating that spontaneous surface oxidation had occurred on the Pt and PtTi surfaces during being stored in the ambient atmosphere. The generated titanium dioxide was a high surface energy material with terminal Ti–OH groups that were easily bound to water molecules, which also contributed to the exhibited hydrophilic behavior of the PtTi film compared to the Pt sample.

Further, the influence of the rough surface structure upon the wettability of a surface could be taken into account. According to the Wenzel state [34], the inherent wetting state of the surface is enhanced by an increase in roughness, and in the Cassie-Baxter state, the increased hydrophobicity can be predicted to result from increased roughness [35]. For this reason, the effect of the roughness on the wettability should not be neglected.

### 3.6. Transmission Electron Microscopy Investigation

**Figure 9:**
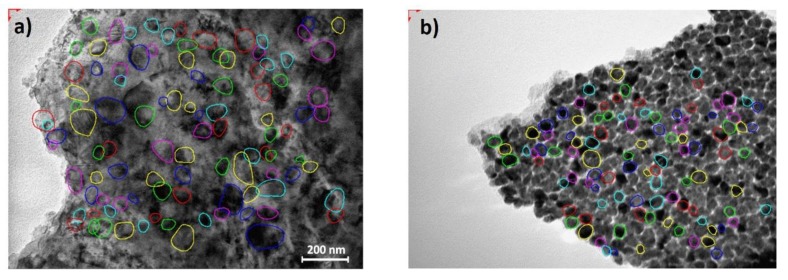
HRTEM images of Pt/Si (**a**) and PtTi/Si (**b**) as the sample start point for measuring the mean size of grain on this sample.

In order to analyze the thin films by TEM, the samples were prepared by the quick method [36] to get small pieces from thin film using a diamond knife to scratch the surface of the film, alcohol as a dispersive medium, and a 400Cu grid covered by a formvar film as support. The structural investigation of the samples was performed by electron diffraction. The HRTEM images showed a uniform film with granular futures, as could be seen in Figure 9.

**Figure 10:**
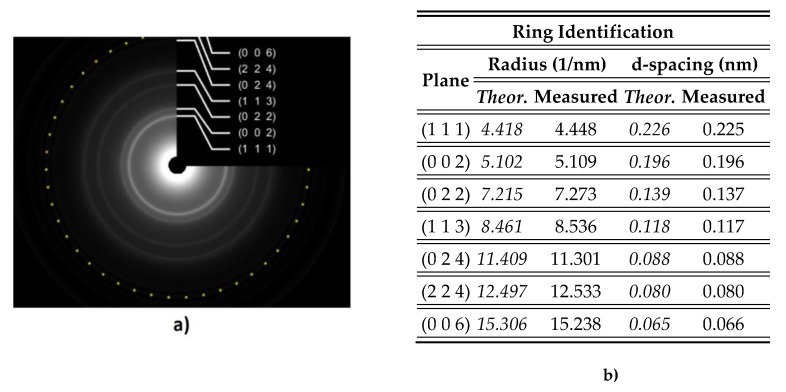
SAED (selected area electron diffraction) investigation for Pt/Si thin films: (**a**) Indexed SAED patterns of Pt/Si film. Inset shows Miller indices for FCC (Face Centered Cubic) Pt; (**b**) Electron diffraction data.

The grain’s mean size for the Pt/Si sample was estimated to be ~4.5 nm (Figure 9). The crystalline structure was revealed by means of electron diffraction [37,38,39]. The rings identified on the SAED pattern could be ascribed to Pt face-centered cubic structure with lattice parameter a = 0.392 nm. Figure 10b shows measured values against theoretical values. 

In the case of PtTi films, the crystalline structure was carried out by means of electron diffraction (Figure 10). 

**Figure 11:**
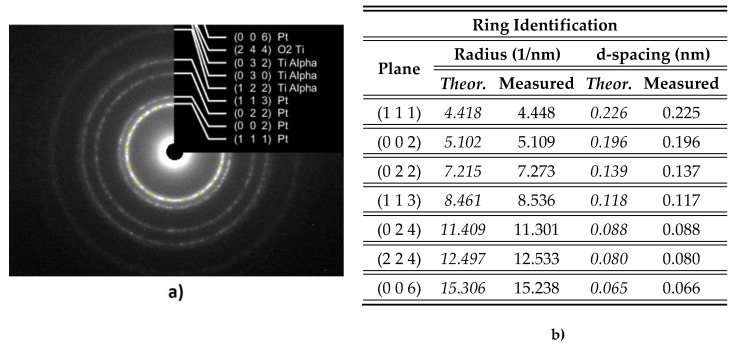
SAED investigation for PtTi/Si thin films: (**a**) Indexed SAED patterns of PtTi/Si. Inset shows Miller indices for FCC Pt tetragonal Ti α and tetragonal TiO_2_–anatase; (**b**) Electron diffraction data.

The rings identified on the SAED pattern could be ascribed to Pt face-centered cubic structure, Ti α, and TiO_2_ anatase phase. Inset shows the Miller indices for FCC (Face Centered Cubic) Pt (a = 0.392 nm), tetragonal Ti α (a = 0.295 nm, c = 0.468 nm), and tetragonal TiO_2_ – anatase phase (a = 0.3785 nm, c = 0.9514 nm). In Figure 11b, the measured values against theoretical values are revealed. The grain’s mean size for the PtTi sample was estimated to be ~ 4.11 nm.

## 4. Conclusions

Pt and PtTi thin films deposited on glass and Si substrates by TVA method showed good uniformity and high purity for critical applications. The SEM images of the films showed a smooth surface without cracks and well-covering the substrate. The surface free energy (SFE) values were the following: for Pt, *γ_Pt/Gl_ =* *13.20 mJ/m^2^,* and for PtTi/Gl, *γ_PtTi/Gl_ =* *42.60 mJ/m^2^*. The grain’s mean size for the Pt/Si sample was estimated to be 4.5 nm, with Pt face-centered cubic structure with lattice parameter *a = 0.392* nm, and, for PtTi sample, the grain’s mean size was 4.11 nm, with Pt face-centered cubic structure, Ti alpha, and TiO_2_ anatase phase. The hardness of PtTi film was substantially higher (5.5 ± 0.5 GPa) than the hardness of Pt film (4.7 ± 0.5 GPa). Based on the mechanical results, we might consider using the deposition TVA technology in some demanding applications that require both the properties of Pt and the mechanical properties of Ti. Therefore, having the data obtained from this study, the quality of the deposited films could be improved by varying both their concentrations and the deposition parameters. 

## Figures and Tables

**Table 1:** EDS elemental analysis for Pt thin film deposited on Si substrate.

Element	Z	Edge	(wt.%)	(Norm. wt.%)	(Norm. at.%)	Error in %
Silicon	14	K	42.89953	39.53	69.59	1.4
Oxygen	8	K	3.74228	5.33	12.39	0.7
Platinum	78	M	53.3582	55.14	18.02	1.9

**Table 2:** EDS elemental analysis for PtTi thin film deposited on a glass substrate.

Element	Z	Edge	(wt.%)	(Norm. wt.%)	(Norm. at.%)	Error in %
Silicon	14	K	32.14497	29.95	55.40	0.50
Oxygen	8	K	3.933436	3.47	3.15	0.50
Platinum	78	M	54.41712	57.01	34.33	0.20
Titanium	22	K	9.504505	9.57	7.06	0.04

**Table 3:** Tribometer experimental data for Pt and PtTi thin films.

Deposited Material	Applied Force (N)	Distance Traveled Before Reaching the Rubstrate (m)	$\bar{\mu }\left(a.u.\right)$	
Pt	0.5	17.5	0.3628	0.38
	1	14.0	0.2885	
	3	10.0	0.4723	
PtTi	0.5	19.0	0.3553	0.35
	1	16.5	0.2688	
	3	12.5	0.4175	

**Table 4:** The roughness parameters of the samples.

Sample	Rp (µm)	Rz (µm)	Rt (µm)	Rc (µm)	Ra(µm)	Rq(µm)
**Pt/Gl**	0.020	0.033	0.210	0.013	0.003	0.007
**PtTi/Gl**	0.131	0.171	0.279	0.077	0.008	0.017

where: Rp — maximum peak height relative to the mean height; Rz — maximum height of the profile; Rt — total height of the profile; Rc — mean height of profile elements; Ra — arithmetical mean height; Rq— root-mean-square deviation (RMS roughness).
